# Barriers to Breast Cancer Screening in Saudi Arabia: A Systematic Review and Meta-Analysis

**DOI:** 10.7759/cureus.65103

**Published:** 2024-07-22

**Authors:** Reem Alsalamh, Faisal A Al-Harbi, Rawan T Alotaibi, Omar N Al-Harbi, Nada Alshahrani, Saleh M Alfadhel, Eyad R Fatani, Abdulaziz Al-Harbi, Razan A Lasloom, Rayan M Alzahrani

**Affiliations:** 1 Surgery, Qassim University, Buraydah, SAU; 2 Medicine, Qassim University, Buraydah, SAU; 3 Medicine, Princess Nourah Bint Abdulrahman University, Riyadh, SAU; 4 Medicine, Vision College, Riyadh, SAU; 5 Medicine, King Abdulaziz University, Jeddah, SAU; 6 Medicine, Najran University, Najran, SAU; 7 Medicine, Al-Baha University, Al-Baha, SAU

**Keywords:** women, saudi arabia, barriers, diagnosis, screening, breast cancer

## Abstract

Breast cancer is a significant public health concern globally, and early detection through screening programs can improve treatment outcomes and reduce mortality rates. However, the uptake of breast cancer screening among women in Saudi Arabia is hindered by various barriers.

This systematic review and meta-analysis aimed to elucidate the barriers to breast cancer screening among women in Saudi Arabia, providing insights into the multifaceted challenges hindering screening uptake and informing tailored interventions and policy recommendations.

A comprehensive literature search was conducted across electronic databases and grey literature sources to identify relevant studies on barriers to breast cancer screening in Saudi Arabia. Studies conducted between 2017 and 2023, employing diverse settings and methodologies, were included in the analysis. Data on the prevalence of barriers, family history of breast cancer, and self-reported breast examination practices were extracted and synthesized.

A total of 14 studies met the inclusion criteria, encompassing diverse populations and methodologies. The included studies predominantly employed cross-sectional survey designs and targeted various populations across different regions of Saudi Arabia. The barriers to breast cancer screening were investigated, revealing concerns such as fear of discovering cancer, embarrassment due to breast-related tests, fear of pain related to clinical examination, and lack of awareness. Additionally, a substantial proportion of participants reported a family history of breast cancer, indicating a significant risk factor for the disease. Self-reported breast examination practices varied among participants, with disparities in screening behaviors observed.

Our review identified fear of diagnosis, embarrassment, and lack of awareness as key barriers to breast cancer screening in Saudi Arabia. Targeted interventions, including education and improved access, are essential to address these challenges and enhance early detection efforts, reducing the burden of breast cancer.

## Introduction and background

Breast cancer represents a significant public health challenge globally, constituting a leading cause of morbidity and mortality among women [1,2]. According to the World Health Organization (WHO), breast cancer is the most prevalent cancer among women worldwide, with an estimated 2.3 million new cases diagnosed annually and approximately 685,000 deaths attributed to the disease in 2020 alone [2,3]. In 2001, there were 5616 instances of cancer in Saudi Arabia for both genders, and then by 2017, that figure had risen by 147.4% to 13,893 cases, in which female cases had risen from 2741 in 2001 to 7975 in 2017 (191% increase), whereas only male cases increased from 2875 in 2001 to 5918 in 2017 (105.8% increase) [4]. Beyond its direct health consequences, breast cancer diagnosis and treatment can engender psychological distress, anxiety, depression, and fear of recurrence among patients, highlighting the complex interplay between the physical and psychological dimensions of the disease [5-7].

Breast cancer prevalence varies across regions, with higher incidence rates observed in developed countries [8]. In 2020, there were around 2.3 million new cases, and 685,000 deaths were reported, for example, in Belgium, there are 112.3 cases per 100,000 people and Iran at 35.8 and mortality rates seen in Fiji at 41 deaths per 100,000 people and South Korea at 6.4 [8,9]. The peak age of breast cancer is over 10 years earlier in some Asian and African countries compared to European or American countries, and as noticed, from 2000 to 2012, breast cancer incidence increased in China and South Korea but decreased in the United States. Also, from 2000 to 2015, the mortality rates rose in China and South Korea [8].

These statistics underscore the need for effective prevention and control strategies. In addition to its high incidence, breast cancer is associated with significant morbidity and mortality, underscoring the urgent need for effective prevention and control strategies [9].

Breast cancer is a multifactorial disease influenced by a combination of genetic, hormonal, environmental, and lifestyle factors [10]. While certain risk factors such as age, gender, and family history are non-modifiable, others, including obesity, alcohol consumption, hormone replacement therapy, and reproductive factors, are potentially modifiable through behavioral and lifestyle modifications [1,11,12]. Understanding these risk factors is crucial for risk stratification, early detection, and targeted interventions aimed at reducing breast cancer incidence and mortality.

Breast cancer screening encompasses various modalities, including mammography, clinical breast examination (CBE), and breast self-examination (BSE), aimed at detecting breast cancer at early, asymptomatic stages when treatment is most effective [13]. Screening plays a pivotal role in reducing breast cancer-related mortality by enabling timely diagnosis and intervention, thus improving treatment outcomes and survival rates [14,15]. Given its potential to identify cancerous lesions before they become clinically apparent, screening is widely advocated as a cornerstone of breast cancer control strategies. In Saudi Arabia, breast cancer represents a significant health burden, with rising incidence rates and associated mortality [4]. Despite efforts to promote breast cancer awareness and screening, the uptake of screening services among Saudi women remains suboptimal [16-18]. Several factors contribute to this phenomenon, including sociocultural norms, limited access to healthcare services, inadequate knowledge and awareness about breast cancer and screening modalities, and fear or stigma associated with cancer diagnosis and treatment [18-20]. As a result, many women forego regular screening, leading to delayed diagnosis and poorer treatment outcomes. Addressing these barriers is imperative to improve screening uptake, facilitate early detection, and reduce the burden of breast cancer in Saudi Arabia. This systematic review and meta-analysis aim to elucidate the barriers to breast cancer screening in Saudi Arabia, offering insights crucial for policymakers, healthcare providers, and researchers to enhance screening uptake and ultimately reduce the burden of breast cancer in the region.

Despite the proven effectiveness of early detection through screening in reducing breast cancer-related mortality [21], the uptake of breast cancer screening remains suboptimal in Saudi Arabia [22,23]. Studies have consistently indicated low rates of mammography utilization and adherence to CBE guidelines among Saudi women, contributing to delayed diagnoses and poorer prognoses [22,23]. Various factors underpin this phenomenon, ranging from individual-level barriers such as lack of awareness and knowledge about breast cancer and screening modalities to systemic challenges such as limited access to healthcare services, cultural stigmatization, and societal norms surrounding women's health issues. Addressing these barriers is imperative to improve breast cancer outcomes and reduce mortality rates among Saudi women.

Understanding the barriers to breast cancer screening in Saudi Arabia is of paramount importance for several reasons. Firstly, Saudi Arabia has witnessed demographic and epidemiological transitions characterized by an increasing burden of non-communicable diseases, including breast cancer, necessitating tailored preventive strategies. Secondly, cultural and religious norms significantly influence health-seeking behaviors and perceptions of illness in Saudi society, potentially exacerbating barriers to breast cancer screening. Thirdly, timely detection through screening is crucial for improving breast cancer outcomes, emphasizing the urgency of addressing impediments to screening uptake. By identifying and addressing these barriers, this study endeavors to contribute to the development of targeted interventions and policies aimed at promoting breast cancer screening and reducing mortality rates among Saudi women.

The primary aim of this systematic review and meta-analysis is to identify and synthesize the existing evidence on the barriers to breast cancer screening among women in Saudi Arabia. By consolidating findings from diverse studies, this research seeks to provide a comprehensive understanding of the multifaceted challenges hindering screening uptake in the Saudi context. Moreover, this study aims to explore variations in barrier prevalence across different demographic groups, geographic regions, and socioeconomic strata to inform the development of tailored interventions and policy recommendations.

## Review

Methodology

This methodology section outlines the systematic approach adopted for conducting the review and meta-analysis on barriers to breast cancer screening in Saudi Arabia.

Literature Search Strategy

A comprehensive search strategy was developed to identify relevant studies from electronic databases, including PubMed, Scopus, Embase, and Web of Science. The search strategy incorporated Medical Subject Headings (MeSH) terms and keywords related to breast cancer screening and barriers. Boolean operators (AND, OR) were used to combine search terms effectively. The search strategy was tailored to each database's syntax and was performed without language restrictions. Grey literature sources, such as conference proceedings and dissertations, will also be searched to minimize publication bias.

Study Selection Criteria

Studies were included based on the following criteria:

Population: Studies involving women residing in Saudi Arabia

Intervention/exposure: Any form of breast cancer screening, including mammography, CBE, or BSE

Outcome: Identification of barriers to breast cancer screening, including individual, sociocultural, and healthcare system-related factors

Study design: Observational studies (cross-sectional, cohort, case-control) and qualitative studies reporting barriers to breast cancer screening

Publication type: Peer-reviewed articles published in academic journals

Exclusion criteria: The following are the exclusion criteria: (a) studies not conducted in Saudi Arabia, (b) studies not focusing on barriers to breast cancer screening, (c) studies lacking primary data or relevant outcomes, (d) studies published in languages other than English, (e) studies not available in full-text format, and (f) review articles, editorials, commentaries, and conference abstracts.

Study Selection Process

**Figure 1:**
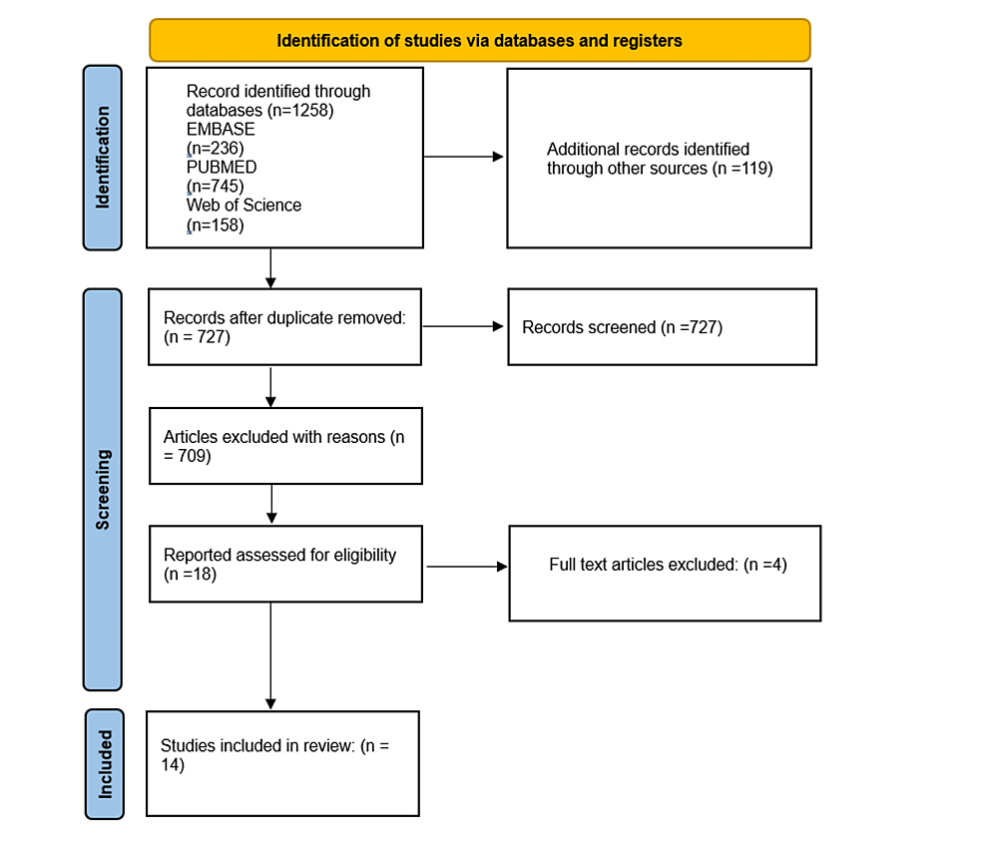
The PRISMA figure showing the steps in choosing the studies for systematic review PRISMA: Preferred Reporting Items for Systematic Reviews and Meta-Analyses

Two independent reviewers conducted the initial screening of titles and abstracts retrieved from the literature search to identify potentially relevant studies. Full-text articles were obtained for further assessment if they meet the inclusion criteria or if there is uncertainty regarding eligibility. Any discrepancies between reviewers were resolved through discussion or consultation with a third reviewer. The Preferred Reporting Items for Systematic Reviews and Meta-Analyses (PRISMA) flow diagram was used to illustrate the study selection process and reasons for exclusion (Figure 1).

Data Extraction

A standardized data extraction form was developed to systematically extract relevant information from included studies. Data extraction will include study characteristics (author, year of publication, study design), participant demographics (age, socioeconomic status), screening methodologies, reported barriers to breast cancer screening, and key findings. Data extraction was conducted independently by two reviewers, with discrepancies resolved through consensus or consultation with a third reviewer.

Data Synthesis and Analysis

A narrative synthesis was conducted to summarize findings from included studies, elucidating common themes and variations in reported barriers to breast cancer screening. Subgroup analyses were performed based on demographic factors (age, socioeconomic status), geographic regions, and screening modalities where feasible. Meta-analysis was conducted if appropriate, utilizing random-effects models to estimate pooled prevalence rates of identified barriers. Sensitivity analyses will be performed to explore sources of heterogeneity and assess the robustness of the results.

Bias Assessment

The Strengthening the Reporting of Observational Studies in Epidemiology (STROBE) checklist was used to assess the bias risk of the included studies (Table 1). It consists of a list of items that should be included in reports of observational studies to ensure transparency and improve the quality of reporting. While the STROBE checklist primarily focuses on reporting quality, it can indirectly help assess the risk of bias by highlighting potential sources of bias if they are not adequately addressed in the study.

**Table 1:** Bias risk assessment of the included studies using the STROBE checklist STROBE: Strengthening the Reporting of Observational Studies in Epidemiology

Study reference	Title and abstract	Introduction	Methods	Results	Discussion	Overall assessment
Abdel-Aziz et al. [16]	Yes	Yes	Yes	Yes	Yes	Low
AlAbdulkader et al. [17]	Yes	Yes	Yes	Yes	Yes	Low
Bakarman et al. [18]	Yes	Yes	Yes	Yes	Yes	Low
Alanazi et al. [20]	Yes	Yes	Yes	Yes	Yes	Low
Alshammari et al. [22]	Yes	Yes	Yes	Yes	Yes	Low
Alenezi et al. [24]	Yes	Yes	Yes	Yes	Yes	Low
Abdel-Salam et al. [25]	Yes	Yes	Yes	Yes	Yes	Low
Al-Zalabani et al. [26]	Yes	Yes	Yes	Yes	Yes	Low
Alshahrani et al. [27]	Yes	Yes	Yes	Yes	Yes	Low
Al-Wassia et al. [28]	Yes	Yes	Yes	Yes	Yes	Low
Eid et al. [29]	Yes	Yes	Yes	Yes	Yes	Low
Soliman El-Hosary et al. [30]	Yes	Yes	Yes	Yes	Yes	Low
Heena et al. [31]	Yes	Yes	Yes	Yes	Yes	Low
Mehmood et al. [32]	Yes	Yes	Yes	Yes	Yes	Low

Results

The systematic review and meta-analysis aimed to elucidate the barriers to breast cancer screening among women in Saudi Arabia, providing insights into the multifaceted challenges hindering screening uptake and informing tailored interventions and policy recommendations. A comprehensive literature search across electronic databases and grey literature sources yielded a total of 14 relevant studies, encompassing diverse settings and methodologies.

The included studies, conducted between 2017 and 2023, predominantly employed a cross-sectional survey design, targeting various populations across different regions of Saudi Arabia. The sample sizes ranged from 127 to 3048 participants, with a total of 9014 individuals included in the analysis. The majority of participants were Saudi nationals, with ages varying across studies, reflecting a broad demographic spectrum. Family history of breast cancer, healthcare worker inclusion, mammography practices, and levels of knowledge about breast cancer screening varied among the studies, providing a nuanced understanding of the factors influencing screening behavior (Table 2).

**Table 2:** General characteristics of the included studies NA: no information is available

Author	Year of publication	Study design	Setting	City/region	Number of the sample	Nationality (Saudi)	Age (mean±SD, range, median)	Family history of breast cancer	Including healthcare worker	Practice of mammogram	Knowledge		
											High	Moderate	Low
Abdel-Aziz et al. [16]	2022	Cross-sectional survey	One general hospital, one tertiary care hospital, and all the primary health centers from the Aljouf region	Aljouf Province	414	284	31.17±6.04, NA, NA	56	Yes	202	93	120	201
AlAbdulkader et al. [17]	2023	Cross-sectional survey	Public places like parks, shopping malls, and masjids	Aljouf Province	400	NA	NA, 40-69, NA	49	No	NA	175	125	100
Bakarman et al. [18]	2023	Cross-sectional survey	Online distribution	Eastern Province	973	835	NA, 40-65, NA	190	No	476	NA	NA	NA
Alanazi et al. [20]	2018	Cross-sectional survey	Primary health centers	Al Hassa	816	NA	43.8±6.6, NA, NA	154	No	132	NA	NA	NA
Alshammari et al. [22]	2023	Cross-sectional survey	Online distribution	Jeddah	328	NA	NA, 20-45, 50	152	No	62	NA	NA	NA
Alenezi et al. [24]	2020	Cross-sectional survey	King Saud University	Riyadh	229	210	NA, <35-46, NA	57	No	43	NA	NA	NA
Abdel-Salam et al. [25]	2020	Cross-sectional survey	Primary health centers	Aljouf Province	423	96	49.12±6.98, 41-75, NA	96	No	NA	NA	NA	NA
Al-Zalabani et al. [26]	2018	Cross-sectional survey	Primary health centers	Madinah	465	352	34.9±12.2, 15-82, NA	82	No	129	18	159	288
Alshahrani et al. [27]	2020	Cross-sectional survey	Maternity and children's hospitals, primary health centers, and King Khalid Hospital	Najran	493	425	NA, 30-50, NA	67	No	NA	85	182	226
Al-Wassia et al. [28]	2017	Cross-sectional survey	Online distribution and at schools and malls	Five main geographic regions of Saudi Arabia	3048	3048	NA, 41-60, NA	828	No	1227	771	1297	1143
Eid et al. [29]	2021	Cross-sectional survey	KSA's Taif University	Taif	478	NA	NA, 18-25, NA	NA	No	NA	NA	NA	NA
Soliman El-Hosary et al. [30]	2021	Cross-sectional survey	Shaqra University	Shaqraa	400	NA	NA, NA, NA	NA	No	NA	NA	NA	NA
Heena et al. [31]	2019	Cross-sectional survey	King Fahad Medical City (KFMC)	Riyadh	420	NA	34.7±8.3, NA, NA	113	Yes	295	5	104	281
Mehmood et al. [32]	2021	Cross-sectional survey	Public sector hospital	Hail	127	NA	46±2, 20-70, NA	29	Yes	NA	23	NA	NA
Total					9014	5250 (58.2%)		1873 (20.7%)		2566 (28.5%)			

The barriers to breast cancer screening in Saudi Arabia were investigated across 14 studies, revealing various concerns and obstacles among participants. Among the identified barriers, fear of discovering cancer and embarrassment due to breast-related tests were the most commonly reported concerns, with 85.7% and 78.6% of studies highlighting this issue, affecting 15.3% and 10.3% of the total participants, respectively. In addition, fear of pain related to clinical examination followed closely, with 64.3% of studies reporting this barrier, impacting 10.3% of participants, and fear of not knowing the procedure was reported in 64.3 % of the studies impacting 19.6% of the participants. Moreover, apprehension regarding radiation exposure also emerged as a prevalent concern with 50.0% of studies highlighting this issue, affecting 8.2% of the total participants, as well as lack of awareness (35.7% of studies, 7.0% of participants). Additionally, concerns such as the perceived lack of importance of mammograms and the belief that cancer has no cure were also notable barriers reported across multiple studies. Financial/work constraints, lack of time, and lack of female healthcare professionals were identified as relatively less common but still significant barriers (Table 3).

**Table 3:** Barriers to breast cancer screening in Saudi Arabia

Barrier	Number of studies	Percent of reported studies	Number of participants	Percent of participants according to the total number of reported barriers	Percent of participants according to the total sample
Fear to discover cancer	12	85.7%	2054	15.3%	22.8%
Embarrassment due to breast-related tests	11	78.6%	1376	10.3%	15.3%
Fear of not knowing the procedure	9	64.3%	1767	13.2%	19.6%
Fear of pain related to clinical examination	9	64.3%	1376	10.3%	15.3%
Apprehension regarding radiation exposure	7	50.0%	1104	8.2%	12.2%
Mammogram is not important	6	42.9%	1907	14.2%	21.2%
Lack of awareness	5	35.7%	945	7.0%	10.5%
Lack of female healthcare professionals	5	35.7%	379	2.8%	4.2%
Cancer has no cure	4	28.6%	669	5.0%	7.4%
Lack of time	4	28.6%	677	5.0%	7.5%
Screening for breast cancer is not worthwhile	3	21.4%	157	1.2%	1.7%
The test may be rejected by the family/ashamed-shy to uncover my breasts	3	21.4%	263	2.0%	2.9%
Financial/work constraints	2	14.3%	592	4.4%	6.6%
No family history of breast cancer	1	7.1%	154	1.1%	1.7%

**Figure 2:**
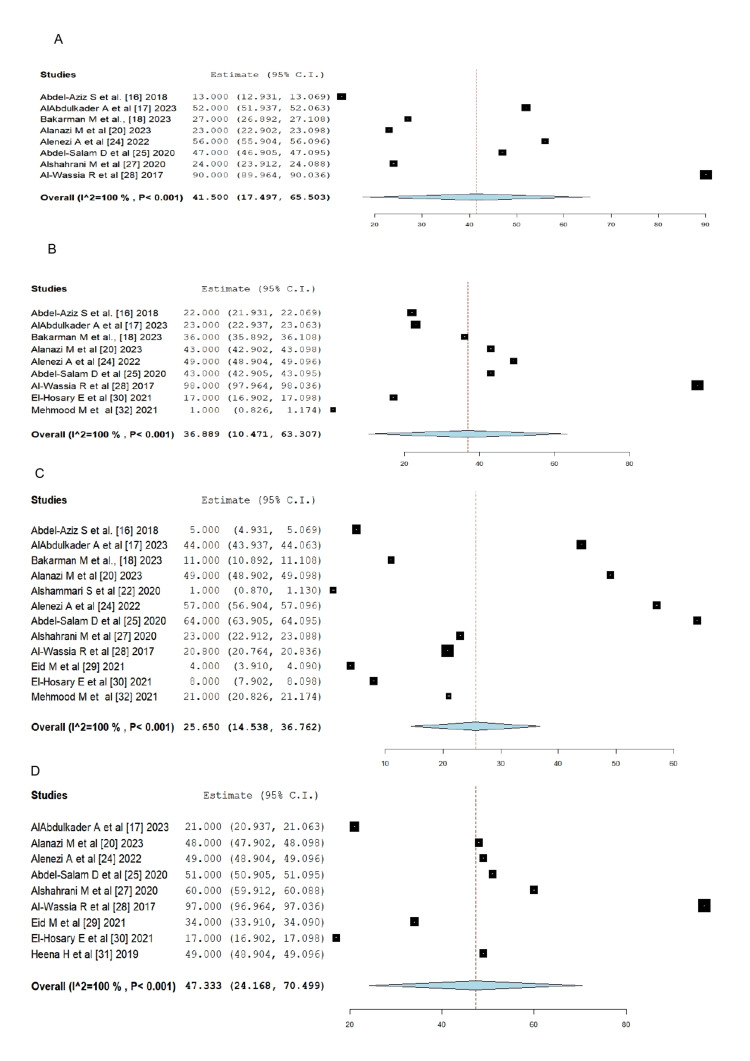
Forest plot of the prevalence of different barriers reported by the participants in different studies (A) Fear of pain related to clinical examination [16-18,20,24,25,27,28] (B) Embarrassment due to breast-related tests [16-18,20,24,25,28,30,32] (C) Fear to discover cancer [16-18,20,22,24,25,27-30,32] (D) Fear of not knowing the procedure [17,20,24,25,27-31]

The meta-analysis was conducted among the studies for each barrier reported as reported in Figure 2. It was found that among eight studies reporting fear of pain related to clinical examination, the prevalence was 41.5% (95% CI: 17.497-65.503) with i2=100% and P=0.0001. In addition, the prevalence of embarrassment due to breast-related tests among the participants of nine studies was 36.89% (95% CI: 9.72-64.05), and among 12 studies, the prevalence of fear to discover cancer was 31.25% (95% CI: 10.22-52.28), and among nine studies, the prevalence of fear of not knowing the procedure was 47.33% (95% CI: 21.13-73.53).

Discussion

Breast cancer screening is crucial for early detection and improved treatment outcomes, yet the uptake of screening programs in Saudi Arabia faces significant barriers. Our systematic review and meta-analysis aimed to shed light on these barriers, drawing insights from 14 studies conducted in the country between 2017 and 2023.

Among the barriers identified, fear of discovering cancer emerged as a predominant concern, with 85.7% of studies reporting this issue. This finding aligns with existing literature highlighting fear and anxiety surrounding cancer diagnosis as major deterrents to screening participation [33-35]. The fear of confronting a potentially life-threatening illness can lead to avoidance behaviors, impacting screening uptake among women in Saudi Arabia [36,37]. Embarrassment due to breast-related tests was another commonly reported barrier, highlighted in 78.6% of studies. This sentiment resonates with cultural norms and modesty considerations prevalent in Saudi society, where discussions and examinations related to intimate body parts can evoke discomfort [38,39]. Addressing cultural sensitivities and providing a supportive environment for screening can help alleviate embarrassment and encourage participation.

Fear of pain related to clinical examination was reported in 64.3% of studies, indicating a significant concern among Saudi women. This finding underscores the importance of patient education and awareness campaigns to dispel misconceptions about the discomfort associated with breast cancer screening procedures [40-42]. By emphasizing the minimal discomfort and potential life-saving benefits of early detection, healthcare providers can mitigate fears and encourage screening adherence. Similarly, fear of not knowing the procedure was highlighted in 64.3% of studies, suggesting a lack of understanding or awareness about the screening process among participants. This barrier emphasizes the need for clear and accessible information about breast cancer screening, including details about the procedure, its purpose, and its potential outcomes [43]. Enhancing health literacy and promoting open communication between healthcare providers and patients can empower women to make informed decisions about their health [44,45].

Apprehension regarding radiation exposure emerged as a prevalent concern, reported in 50.0% of studies. While mammography remains a gold standard for breast cancer screening, concerns about radiation exposure may deter some women from undergoing screening [46]. Educating women about the minimal radiation dose associated with mammograms and emphasizing the benefits of early detection in outweighing potential risks can help address this barrier [47].

Lack of awareness was identified in 35.7% of studies, indicating gaps in knowledge about breast cancer screening guidelines, benefits, and available services. This finding underscores the importance of community outreach programs and educational initiatives to increase awareness about breast cancer and screening options [48,49]. Collaborative efforts between healthcare providers, community leaders, and media outlets can disseminate accurate information and promote proactive health-seeking behaviors.

In addition to these commonly reported barriers, several studies highlighted concerns such as the perceived lack of importance of mammograms and the belief that cancer has no cure. These findings underscore the influence of cultural beliefs and misconceptions on screening behaviors [50,51]. Addressing misconceptions through culturally sensitive health promotion strategies and tailored interventions can help improve screening rates and early detection efforts. Financial/work constraints, lack of time, and lack of female healthcare professionals were identified as relatively less common but still significant barriers. These findings highlight the broader systemic challenges and access barriers faced by women in Saudi Arabia, particularly those from marginalized communities. Policy interventions aimed at reducing financial barriers, expanding access to screening facilities, and increasing the representation of female healthcare professionals can enhance equitable access to breast cancer screening services.

In addition to exploring barriers, our analysis also revealed insights into the prevalence of family history of breast cancer and self-reported breast examination practices among women in Saudi Arabia. Our findings indicate that a substantial proportion of women have a family history of breast cancer, with 1873 individuals (20.7%) across the included studies reporting a positive family history. This prevalence underscores the significance of genetic predisposition as a risk factor for breast cancer among Saudi women [52]. Understanding the prevalence of family history can inform risk-stratified screening approaches and facilitate early detection efforts among high-risk individuals. Targeted screening and genetic counseling services for women with a family history of breast cancer are essential for early diagnosis and proactive management.

Regarding self-reported breast examination practices, our analysis revealed disparities in screening behaviors among women in Saudi Arabia. A considerable number of individuals, totaling 2566 (28.5%) across the included studies, reported practicing mammograms. While this indicates a notable proportion of women engaging in recommended screening practices, disparities in screening uptake and awareness persist. Some women may lack access to screening facilities or face barriers such as financial constraints or cultural stigma, which can impact screening behaviors [26]. Promoting regular breast self-examination and enhancing awareness about the importance of early detection strategies are crucial steps in improving screening rates and reducing breast cancer mortality rates in Saudi Arabia.

Limitations of the study

Despite the valuable insights gleaned from our systematic review and meta-analysis, several limitations should be acknowledged. Firstly, the included studies exhibited heterogeneity in terms of population characteristics and measurement methods, which may have introduced variability and bias into the findings. Additionally, the reliance on self-reported data in some studies may have led to recall bias and underestimation or overestimation of certain variables, such as screening practices and barriers. Furthermore, the majority of included studies were cross-sectional in nature, precluding the establishment of causal relationships and longitudinal assessment of screening behaviors over time. Future research employing prospective cohort designs and standardized measurement tools is warranted to provide more robust evidence on barriers to breast cancer screening and their impact on screening uptake among women in Saudi Arabia.

## Conclusions

Our systematic review and meta-analysis shed light on the multifaceted barriers hindering breast cancer screening uptake among women in Saudi Arabia. Fear of cancer diagnosis, embarrassment, fear of pain, lack of awareness, and concerns about radiation exposure were among the predominant barriers identified, reflecting the intricate interplay of individual, cultural, and systemic factors shaping screening behaviors. Additionally, disparities in screening practices and awareness persist, highlighting the need for targeted interventions to improve access and promote proactive health-seeking behaviors.

Overall, our findings underscore the complex interplay of individual, cultural, and systemic factors influencing breast cancer screening uptake among women in Saudi Arabia. Addressing these barriers requires a multifaceted approach, encompassing targeted education, community engagement, policy reforms, and healthcare system improvements. Culturally sensitive health promotion strategies, enhanced patient education, and increased accessibility to screening facilities are essential components of this approach. By addressing barriers and promoting proactive screening behaviors, we can enhance early detection efforts and ultimately reduce the burden of breast cancer in Saudi Arabia.
